# Transplantation of autologous bone marrow pre-loaded *ex vivo* with oncolytic myxoma virus is efficacious against drug-resistant Vk*MYC mouse myeloma

**DOI:** 10.18632/oncotarget.28205

**Published:** 2022-03-03

**Authors:** Nancy Y. Villa, Masmudur M. Rahman, Joseph Mamola, Meaghen E. Sharik, Ana Lemos de Matos, Jacquelyn Kilbourne, Kenneth Lowe, Juliane Daggett-Vondras, Julia D'Isabella, Elizabeth Goras, Marta Chesi, P. Leif Bergsagel, Grant McFadden

**Affiliations:** ^1^Biodesign Institute, Center for Immunotherapy, Vaccines and Virotherapy (CIVV), Arizona State University, Tempe, AZ 85281, USA; ^2^Division of Hematology/Oncology, School of Medicine, Emory University, Atlanta, GA 32322, USA; ^3^Department of Medicine, Mayo Clinic, Scottsdale, AZ 85259, USA

**Keywords:** myxoma virus, multiple myeloma, combination therapy, autologous transplantation, oncolytic virus

## Abstract

Multiple myeloma (MM) is a hematological malignancy of plasma cells that remains incurable despite significant progress with myeloablative regimens and autologous stem cell transplantation for eligible patients and, more recently with T cell redirected immunotherapy. Recently, we reported that *ex vivo* virotherapy with oncolytic myxoma virus (MYXV) improved MM-free survival in an autologous-transplant Balb/c mouse model. Here, we tested the Vk*MYC transplantable C57BL/6 mouse MM model that more closely recapitulates human disease. *In vitro*, the murine bortezomib-resistant Vk12598 cell line is fully susceptible to MYXV infection. *In vivo* results demonstrate: (i) autologous bone marrow (BM) leukocytes armed *ex vivo* with MYXV exhibit moderate therapeutic effects against MM cells pre-seeded into recipient mice; (ii) Cyclophosphamide in combination with BM/MYXV delays the onset of myeloma in mice seeded with Vk12598 cells; (iii) BM/MYXV synergizes with the Smac-mimetics LCL161 and with immune checkpoint inhibitor α-PD-1 to control the progression of established MM *in vivo*, resulting in significant improvement of survival rates and decreased of tumor burden; (iv) Survivor mice from (ii) and (iii), when re-challenged with fresh Vk12598 cells, developed acquired anti-MM immunity. These results highlight the utility of autologous BM grafts armed *ex vivo* with oncolytic MYXV alone or in combination with chemotherapy/immunotherapy to treat drug-resistant MM *in vivo*.

## INTRODUCTION

Multiple myeloma (MM) is the second most common hematological malignancy in the world [1, 2]. MM is characterized by the clonal expansion of malignant plasma cells (PCs) within a permissive bone marrow microenvironment that promotes the survival and proliferation of tumor cells [3–5]. Despite the remarkable progress in MM treatment, resulting in more prolonged disease-free survival, the disease remains incurable. Despite continued advances in new generations of drugs and novel therapies such as CAR-T cells, high dose ablative chemotherapy/radiotherapy along with autologous stem cell transplantation for eligible patients are still standard therapies used to treat myeloma patients [1]. However, the imminent relapse of the disease and its subsequent acquired resistance to existing drug therapies remain major challenges that still need to be overcome in order to assure long-term patient survival.

Recently, we reported compelling experimental evidence indicating that the oncolytic myxoma virus (MYXV) can eliminate minimal residual MM disease in the setting of either allogeneic- [6] or autologous-stem [7] cell transplantation in an immunocompetent Balb/c mouse transplanted with mineral-oil induced plasmacytomas (MOPC)315.BM.dsRed cells [8]. Although the preclinical MOPC315.BM.dsRed myeloma model has been informative for us and others [8], and is well accepted as a preclinical model of MM, the MOPC model has some drawbacks. For example, in addition to the BM, the MM tumors can develop in the spleen and some mice exhibit extramedullary tumors (i.e., solid tumors outside the bone marrow compartment) [4]. Therefore, we aimed to overcome some of these limitations by using a mouse model of MM that more faithfully recapitulates the development, clinical manifestations and localization of the disease observed in human MM patients.

In 2008, Chesi and co-workers reported a murine C57BL/6-derived Vk*MYC MM preclinical model characterized by low proliferation of monoclonal plasma cells (PCs) within the Vk*MYC mice BM and secondary lymphoid organs, and therefore more closely resembling the feature of monoclonal gammopathy of undetermined significance (MGUS)/MM. Like in humans, this murine model produces abnormal high level of serum monoclonal Ig antibodies resulting in a detectable M-spike that can be measured in blood serum, and represents a clonal marker of tumor burden [9]. In terms of clinical manifestations, this model shows reduced levels of hemoglobin and bone mineral density (BMD), and MM-like kidney damage, which surrogates the clinical manifestation of the human disease [9]. Furthermore, this preclinical model has been used to accurately predict the chemotherapeutic effects of different anti-MM drugs [10]. Another peculiarity of the Vk*MYC model is the reproduction of cross talk between clonal PCs and the tumor microenvironment [10]. Upon harvesting serial passages of bortezomib (BOR)-resistant tumors from aged Vk*MYC mice, two independent BOR-resistant myeloma cell lines were generated and designated the transplantable BOR-resistant Vk12598 and the multidrug-resistant Vk12563 cells [10]. These cell lines can be transplanted to younger syngeneic C57BL/6 mice allowing for myeloma engraftment and aggressive disease development.

Here we demonstrated that murine BOR-resistant Vk12598 MM cells are fully susceptible to MYXV infection and oncolysis *in vitro*. On the other hand, *in vivo* data indicate that although virotherapy with free MYXV (i.e., un-armed vMyx-M135KO or armed human TNF expressing vMyx-hTNF recombinant constructs) delays the onset of MM disease, this monotherapy treatment was insufficient to eliminate or control the eventual progression of the disease. In contrast, transplantation with autologous BM cells *ex vivo* pre-loaded with either un-armed or TNF-armed MYXV improved survival rates in a fraction of recipient mice pre-seeded with Vk12598 cells. Furthermore, combination therapy with the therapeutic alkylating agent cyclophosphamide (Cy), which, by itself, induces a partial response in patients with MM [11, 12] along with autologous BM *ex vivo* pre-loaded with either un-armed vMyx-M135KO, or vMyx-hTNF only delayed the onset of established MM disease inducing only a moderate improvement of survival rates that was not statistically significance as compared to single treatment with Cy. In striking contrast, we found that treatment of recipient mice bearing Vk12598 cells with autologous bone marrow (BM) cells *ex vivo* treated with either un-armed or human TNF-armed MYXV virus constructs and in combination with the second mitochondrial-derived activator of caspases (Smac)-mimetics compound LCL161 and the immune checkpoint inhibitor (ICI) α-PD-1 resulted in long-term survival and decrease of tumor burden in recipient mice. Importantly, when survivor mice from either of the last two cohorts were re-challenged with fresh Vk12598 cells, they were now resistant to the disease, suggesting that these mice had now acquired anti-myeloma immunity. Results from this study, demonstrate the therapeutic potential of using either un-armed or hTNF-armed MYXV in combination with auto-BM transplantation and chemo/immunotherapy to control the progression of BOR-resistant MM disease.

## RESULTS

### Murine Vk12598 MM cells are susceptible to MYXV binding and infection *in vitro*

**Figure 1:**
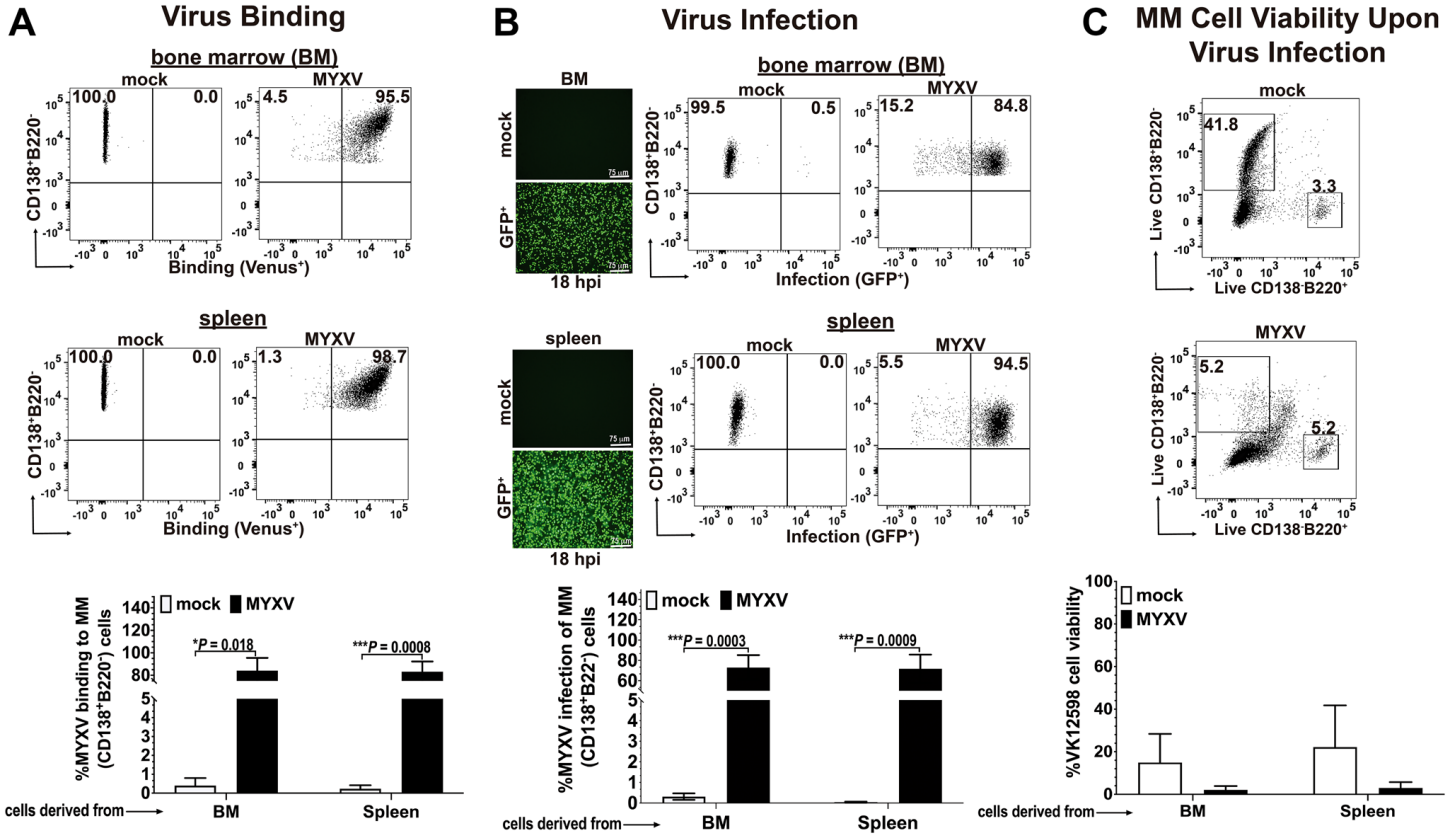
Murine BOR-resistant Vk12598 myeloma cells are susceptible to MYXV binding and infection *in vitro*. (**A**) Flow cytometry 2D-plots showing the levels of vMyx-M093L-Venus binding to fresh BOR-resistant Vk12598 MM cells derived from bone marrow and spleen (top and middle panels, respectively). The percentage of virus binding to Vk12598 cells derived from either BM, or spleen are summarized in (A)-bottom panel. (**B**), Fluorescent micrographs and flow cytometry 2D-plots showing the levels of infection of Vk12598 cells (top and middle panels). The percentages of infection (GFP expression from vMyx-GFP) of Vk12598 MM cells derived from BM or spleen after 18 h of incubation at 37°C is shown in bottom panel. (**C**) Flow cytometry 2D-plots of a representative experiment showing the percentage of MM cells remaining (CD138^+^B220^−^) upon mock-treatment or infection with vMyx-GFP after 18 h at 37°C (top and middle panels, respectively). The percentages of MM cell viability after mock- or MYXV infection is shown in bottom panel. Plots represent the mean ± standard deviation (SD) of at least 3 independent experiments. *P* values are reported as statistically significant when ^*^ *P* < 0.05, ^***^ *P* 0.001.

Similar to primary human myeloma cells, BOR-resistant Vk12598 myeloma cells are dependent on the native BM microenvironment to grow and proliferate [10]. Therefore, these murine Vk12598 cells cannot grow *in vitro* because of their very low proliferating index [10]. Although, we could not perform long-term *in vitro* experiments with these murine MM cells because of their imminent cell death in culture, we could perform short-time *in vitro* experiments lasting 1 h or maximum 18 h at the most in order to determine the susceptibility of these BOR-resistant Vk12598 myeloma cells to MYXV binding and infection. In brief, 1 × 10^6^ Vk12598 cells isolated from BM or splenocytes derived from C57BL/6 mice pre-seeded for 4 weeks were used to determine virus binding or infection. For virus binding assays, fresh Vk12598 from either bone marrow (BM) or the spleen compartment were exposed to vMyx-M093L-Venus (a wild-type MYXV that expresses Venus-tagged M093 protein as virion component) at a multiplicity of infection (MOI) of 10 for 1 h at 4°C, to allow virus binding to the cell surface. After 1 h the unbound virus was washed twice with cold 1x-PBS + 5% FBS. The levels of Venus-tagged virus binding to MM cells (i.e., CD138^+^Venus^+^) were assessed using flow cytometry (Figure 1A). Also, to monitor active virus infection, fresh 1 × 10^6^ Vk12598 cells were incubated with vMyx-GFP (MYXV that expresses GFP from a constitutive early/late virus promoter) at MOI of 10 for 1 h at 37°C, to allow virus adsorption. After this, cells were incubated overnight (i.e., 18 h) at 37°C to allow virus entry and infection. Infection of MM cells (i.e., CD138^+^GFP^+^) were determined using fluorescent microscopy and the levels of infection were assessed using flow cytometry (Figure 1B). Interestingly, loss in myeloma cell number was evident upon infection with vMyx-GFP for 18 h, as compared to mock control (i.e., with mock: 41.8% vs. with MYXV: 5.2% viability), (Figure 1C). The flow cytometry 2D-plots of a representative experiment are shown in the top panel of Figure 1C. Together, the data shown in Figure 1 demonstrate the susceptibility of BOR-resistant Vk12598 MM cells to MYXV binding and infection. Importantly, even when considering the short life span of these myeloma cells in culture, MYXV infection further abrogated the viability of the Vk12598 cells *in vitro*.

### Autologous bone marrow *ex vivo* loaded with unarmed-MYXV or TNF-armed MYXV delays the onset of MM disease *in vivo* and improves survival rates in mice pre-seeded with BOR-resistant murine Vk12598 myeloma cells

**Figure 2:**
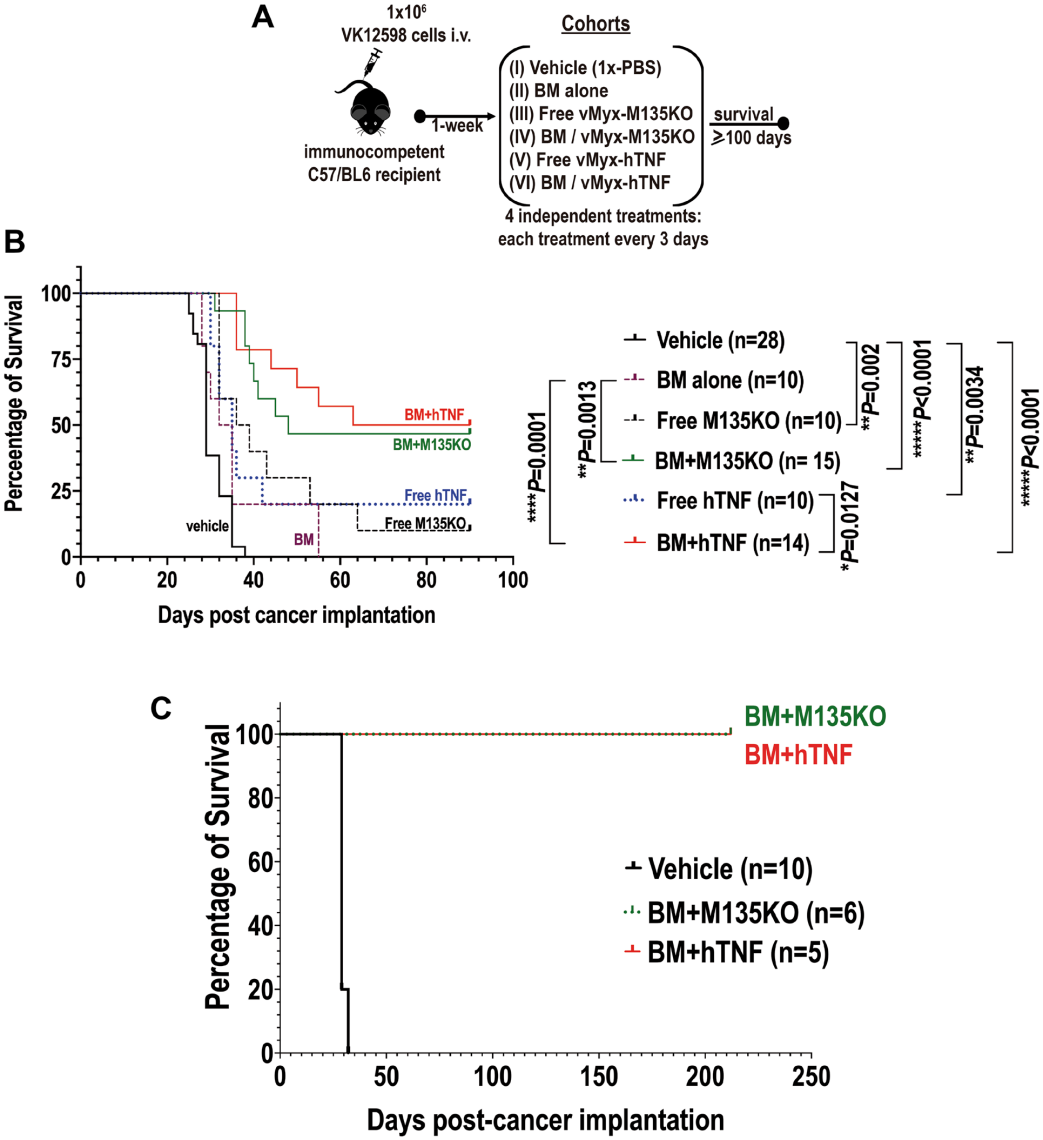
Autologous bone marrow *ex vivo* loaded with MYXV not only resulted in improvement of survival rates of recipient mice pre-seeded with Vk12598 myeloma cells, but also a fraction of these survivor mice became resistant after re-challenging them with myeloma cells. (**A**) Diagram describing the experimental design including the treatment time-line after myeloma implantation. (**B**) Survival curves at day 90 post-cancer implantation, of different cohorts comparing the therapeutic effects of virotherapy with free un-armed vMyx-M135KO virus alone or in combination with BM transplantation versus free armed vMyx-hTNF virus alone or in combination with auto-BM transplantation. (**C**) Survival curves of survivor mice re-challenged with fresh Vk12598 multiple myeloma cells. In panel (C), cohort treated with 1x-PBS was used as a control to demonstrate the susceptibility of the mice seeded with fresh Vk12598 MM cells. The *P* values for log-rank Mantel-Cox survival test, are shown in (B). *P* values are reported as statistically significant when ^**^ *P* 0.01, ^***^ *P* 0.001, ^****^ *P* ≤ 0.0001.

Based on the *in vitro* results shown in Figure 1, we next tested whether virotherapy with MYXV alone or in combination with autologous stem cell transplantation (ASCT) can promote therapeutic effects in mice seeded with BOR-Vk12598 myeloma cells. We also wanted to exploit the therapeutic effects of an un-armed MYXV construct (using vMyx-M135KO) and a transgene-armed MYXV construct vMyx-hTNF (MYXV expressing human TNF) that has been recently shown to be more therapeutic than unarmed MYXV in a murine model of lung metastatic osteosarcoma [13]. To assess this, 8-weeks old immunocompetent recipient C57BL/6 mice were implanted with 1.0 × 10^6^ Vk12598 murine MM cells intravenously (i.v.) via the tail vein. One week after cancer implantation, mice cohorts were treated as follows: vehicle control (i.e., no BM transplant: PBS only) (*n* = 28, cohort I); autologous C57BL/6 BM alone (*n* = 10, cohort II); free un-armed vMyx-M135KO virus construct (*n* = 10, cohort III); autologous BM cells *ex vivo* treated with un-armed vMyx-M135KO virus construct (*n* = 15, cohort IV); free transgene-armed vMyx-hTNF virus construct (*n* = 10, cohort V); autologous BM cells *ex vivo* treated with transgene-armed vMyx-hTNF virus construct (*n* = 14, cohort VI). All the test therapeutic treatments were delivered using the retro-orbital (r.o.) route. Recipient mice from each cohort received four consecutive treatments, each treatment every 3 days as described in Figure 2A. Figure 2B shows the Kaplan-Meier survival curves of these cohorts. The survival was scored at day 90 post-cancer implantation. Although the treatment with free MYXV (either armed or unarmed) slightly delayed the onset of the disease, the therapeutic effects were not statistically significant (i.e., percentage of survival: vehicle control = 0.0%; BM alone = 0.0%, free vMyx-M135KO = 10.0%; free vMyx-hTNF = 20.0%). In contrast, mice treated with autologous BM *ex vivo* treated with unarmed vMyx-M135KO or armed vMyx-hTNF resulted not only in the delay of the onset of the disease but also some therapeutic effects, (i.e., percentage of survival: BM+vMyxM135KO = 46.7%; BM+vMyx-hTNF = 50.0%). However, the survival rates of these two cohorts were not statistically significant from each other, suggesting no functional difference between the un-armed virus vs. the hTNF-expressing armed MYXV construct. The results shown in Figure 2B summarize two independent *in vivo* experiments. In order to determine if cured mice developed acquired anti-tumor immunity to MM disease, survivor mice from our second *in vivo* experiment [i.e., survivor mice treated with either BM+vMyx-M135KO (*n* = 6), or BM+ vMyx-hTNF (*n* = 5)] were re-challenged with 1 × 10^6^ fresh Vk12598 cells. Importantly, these latter mice survived for 212 days after re-challenging with no signs of myeloma disease as compared to mice bearing MM and treated with vehicle control (1x-PBS), (Figure 2C). Taking together, these data indicate that autologous BM *ex vivo* loaded with either un-armed MYXV (vMyx-M135KO) or armed MYXV with human TNF (vMyx-hTNF) had superior therapeutic effects than virotherapy with systemic infusion of either free unarmed or human TNF transgene armed MYXV.

### Combination of cyclophosphamide (Cy) with autologous bone marrow cells *ex vivo* loaded with MYXV delays the onset of Vk12598 multiple myeloma *in vivo*

**Figure 3:**
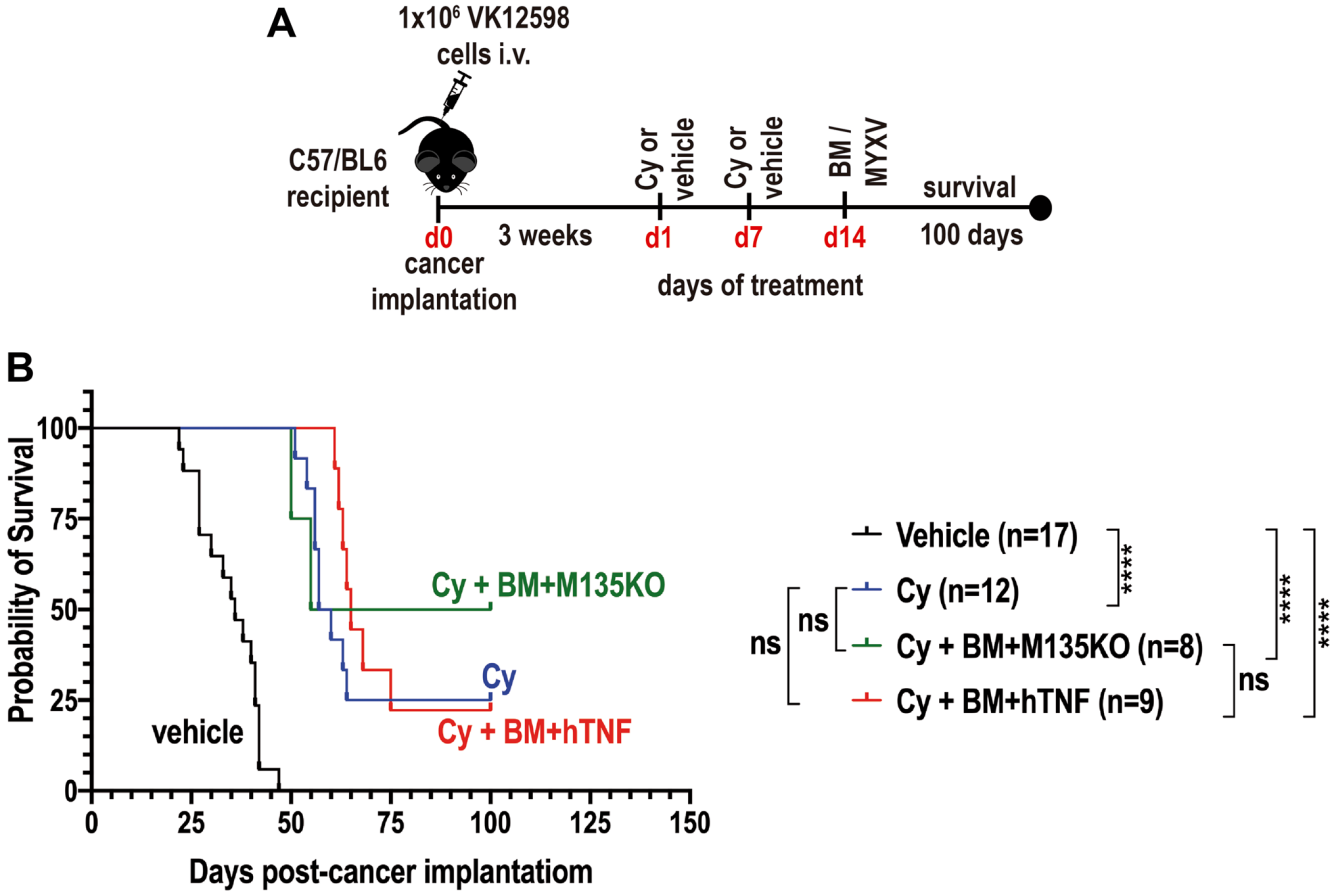
Combination therapy with cyclophosphamide (Cy) and autologous bone marrow (BM) pre-loaded with MYXV delay the onset of MM. (**A**) Diagram describing the experimental design including the treatment time-line after myeloma implantation. (**B**) Survival curves of different cohorts comparing the therapeutic effects of 100 mg/Kg cyclophosphamide (Cy) alone versus Cy followed by transplant with autologous BM pre-loaded with un-armed vMyxM135KO virus versus Cy followed by transplant with autologous BM pre-loaded with armed vMyx-hTNF virus. (B) Survival curves show the number of treated mice (n), survival (in days) from the beginning of cancer implantation, and *P* values for log-rank Mantel-Cox survival test. *P* values are reported as not statistically significant (ns) when *P* > 0.05 or statistically significant when ^****^ *P* ≤ 0.0001.

Because our goal was to explore different therapeutic strategies that effectively target and eliminate resistant MM cells *in vivo*, we decided to exploit combination therapy with the alkylating agent cyclophosphamide (Cy) along with auto-transplantation of BM *ex vivo* treated with un-armed or armed MYXV. Cy is one of the up-front chemotherapeutic agents used to treat patients with MM [11, 12]. Cy has been reported to potentiate T cell redirected therapy and other adaptive immune responses against established tumors by abrogating regulatory T cells and restores T and NK cell functions [14–18]. In addition to this, Cy induces an acute secretory activating phenotype that promotes non-differentiated macrophage (MΦ) infiltration and phagocytosis of tumor cells [12]. Because Cy partially abrogates the progression of MM resulting in tumor reduction [11, 12], we hypothesized that Cy might synergize with autologous BM pre-loaded with MYXV to improve survival of mice bearing established BOR-resistant MM disease. In order to test this hypothesis, 8-weeks old immunocompetent C57BL/6 recipients were injected with 1.0 × 10^6^ Vk12598 MM cells intravenously (i.v.) via the tail vein. Unlike the previous study (Figure 2), in this study myeloma cells were allowed to establish later-stage disease for 3 weeks prior to initiation of therapy as described in Figure 3A. After this, mice were treated with either vehicle control (i.e., 1x-PBS) (*n* = 17, cohort I) via the retro-orbital (r.o.) route, or with 100 mg/Kg Cy alone delivered via the intra-peritoneally (i.p.) route, twice, each dose one week apart (i.e., at days 1 and 7), (*n* = 12, Cohort II) in order to induce minimal residual disease (MRD) (Figure 3A). At day 14 after drug treatment, some of the mice that were pretreated with 100 mg/Kg of Cy were transplanted with either BM *ex vivo* pre-loaded with un-armed vMyx-M135KO (*n* = 8, cohort III), or with BM *ex vivo* pre-loaded with armed vMyx-hTNF (*n* = 9, cohort IV). Treatments with BM *ex vivo* pre-loaded with MYXV were delivered using the retro-orbital (ro) route. The survival curves of these cohorts are shown in Figure 3B. As can be seen, the treatment with Cy alone, or in combination with autologous BM/MYXV resulted in some improvement of survival rates of mice seeded with MM as compared with mice treated with vehicle control (i.e., percentage of survival with vehicle control = 0.0%; Cy alone = 25.0%; Cy + BM/vMyx-hTNF = 22.0%; Cy + BM/vMyxM135KO = 50.0%). However, we could not establish statistical differences among cohorts II, III and IV. We speculate that *in vivo* pretreatment of myeloma cells with Cy can compromise MYXV spread or distribution within the tumor milieu, which ultimately hampers viral oncolysis specifically at high MOIs (i.e., MOI = 10). In fact, it has been reported by others that Cy in combination with low doses of rQNesting34.5, a modified herpes simplex virus-1 (HSV-1), increased survival of established xenograft glioma, as compared to combination of Cy and high doses of rQNesting34.5 [19]. Another possibility is that Cy in combination with high load levels of virus might be very toxic, limiting the therapeutic effects of this combination treatment (i.e., Cy and BM/MYXV) [19]. In addition to this, it is known that in terms of innate immune mechanisms, Cy stimulates antiviral immune defenses. This latter could result in increased antibody-mediated viral neutralization and ultimately clearing the oncolytic virus [20]. Taken together, the results shown in Figure 3B demonstrate that combination therapy with the alkylating agent Cy plus autologous BM transplant *ex-vivo* pre-loaded with MYXV delays the progression of BOR-resistant Vk12598 MM cells. However, we could not establish significant therapeutic benefits in terms of improved survival rates upon treatment of the recipient mice with Cy alone or Cy in combination with BM/M135KO, or BM/hTNF.

### Autologous murine bone marrow cells *ex vivo* loaded with MYXV synergizes with the Smac-mimetics compound LCL161 and the checkpoint inhibitor α-PD-1 against murine BOR-resistant Vk12598 multiple myeloma cells

**Figure 4:**
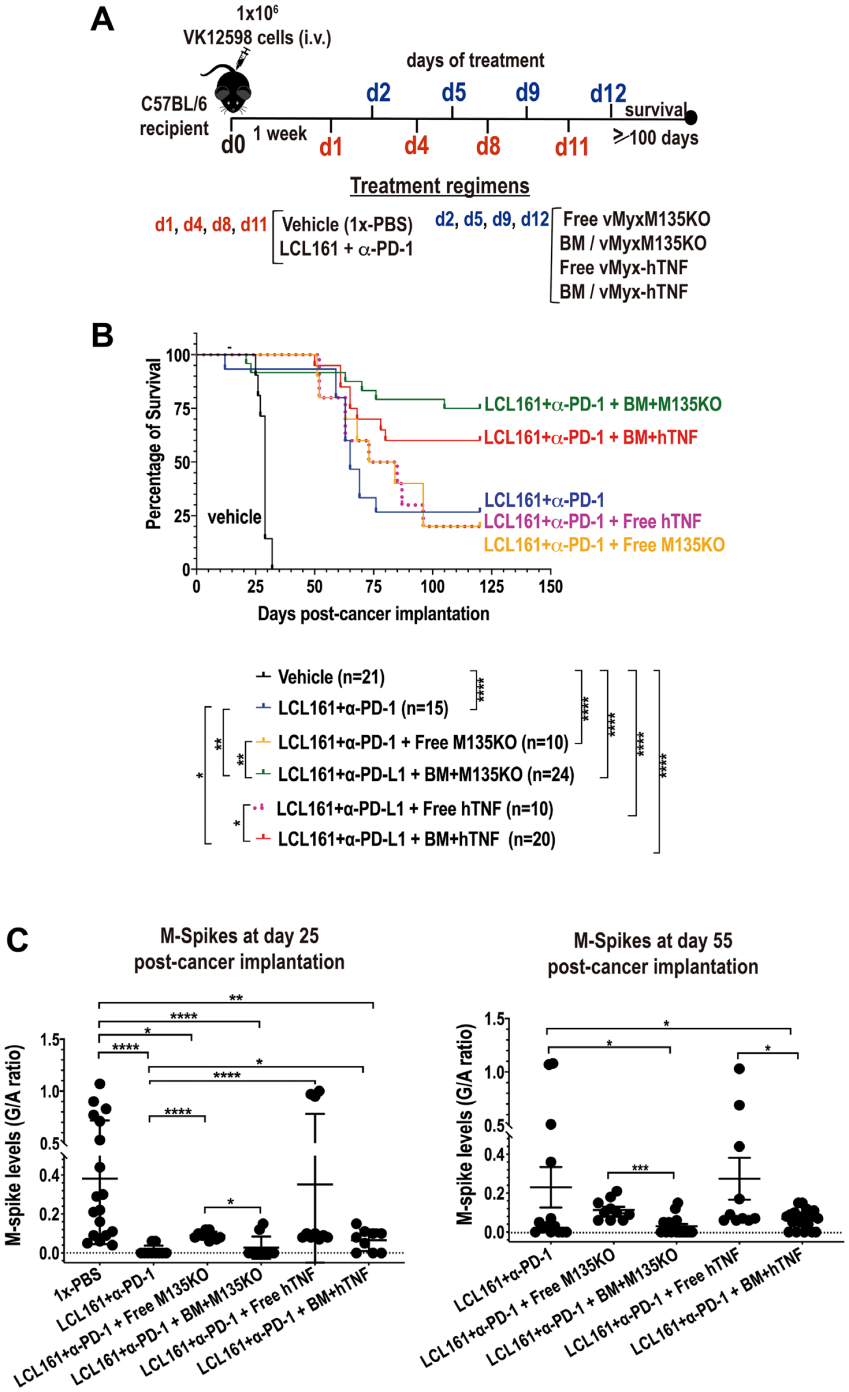
Autologous BM *ex vivo* preloaded with either un-armed MYXV, or with hTNF transgene armed MYXV synergizes with LCL161 + α-PD-1 against BOR-resistant Vk12598 myeloma cells *in vivo*. (**A**) Diagram describing the experimental design including the treatment time-line after myeloma implantation. (**B**) Survival curves of mice treated with LCL161 + α-PD-1 +/− free vMyxM135KO versus free vMyx-hTNF, LCL161 + α-PD-1 + BM *ex vivo* pre-loaded with either vMyxM135KO, or vMyx-hTNF. Survival curves (B) show the number of treated mice (n), survival (in days) from the beginning of cancer implantation, and *P* values for log-rank Mantel-Cox survival test. *P* values are reported as not statistically significant (ns) when *P* > 0.05 or statistically significant when ^*^ *P* < 0.05, ^**^ *P* 0.01, ^***^ *P* 0.001, ^****^ *P* ≤ 0.0001. (**C**) Plots the M-spike levels in sera in response to each indicated treatment regimen at day 25 or at day 55. Student’s *t*-test was used to compare the mean of each M-spike with every other M-spike mean post cancer implantation.

Results shown in Figure 3B suggest that MYXV can be used as adjunct therapy to treat aggressive and BOR-resistant Vk12598 MM cells *in vivo*. Knowing this, we next explored combination therapy with the Smac-mimetics compound LCL161 along with immunotherapy using the immune checkpoint inhibitor (ICI) α-PD-1. Smac-mimetics LCL161 compound has been developed as an antagonist of the cellular inhibitor of apoptosis cIAP-1 and -2 and has been unsuccessfully evaluated in clinical trials for its ability of induce TNF-mediated apoptosis of cancer cells [12, 21]. On the other hand, by activating the non-canonical NFkB pathway, LCL161 has been shown to manipulate the tumor microenvironment inducing immune activation and resulting in long-lasting protection against myeloma progression *in vivo* [12]. Furthermore, combination therapy with LCL161 plus α-PD-1 strikingly improved survival rates of recipient mice bearing MM [12]. Based on these reported results, we decided to investigate sequential treatments with un-armed MYXV or armed MYXV with the hTNF transgene alone or in combination with autologous BM transplant and with LCL161 + α-PD-1 against MM. In brief, eight-weeks old C57BL/6 mice were injected with 1.0 × 10^6^ Vk12598 myeloma cells intravenously (i.v.) via the tail vein. One week after cancer implantation, mice were treated on day 1, 4, 8 and 11 with: 50 mg/kg of LCL161 via the oral gavage (o.g.) route or 10 mg/mL of α-PD-1 intraperitoneally (i.p.), or both LCL161 + α-PD-1. On days 2, 5, 9 and 12, these mice were injected with autologous free viruses or with BM *ex vivo* pre-loaded with either un-armed vMyx-M135KO or with armed vMyx-hTNF via the retro-orbital (r.o.) route. Control mice were treated only with vehicle. Under these experimental conditions, we found that combination of LCL161 + α-PD-1 coupled with autologous BM pre-loaded with either vMyx-M135KO or with vMyx-hTNF significantly improved survival rates of mice bearing Vk12598 myeloma cells as compared with the combination of LCL161 + α-PD-1 with either free vMyx-M135KO or vMyx-hTNF (i.e., percentages of survival upon combination therapy with LCL161 + α-PD-1 followed by: BM/vMyx-M135KO = 75.0%; BM/hTNF = 60.0%; free vMyx-M135KO = 20.0%; free vMyx-hTNF = 20.0%; LCL161 + α-PD-1 = 26.7%). Under these experimental conditions we did not observe increased survival when recipient mice were treated with LCL161 + α-PD-1 only as reported in a former publication using transplantable murine Vk*MYC model [12], Figure 4B. This discrepancy might be attributed to the origin of the mice (Charles River in this case) and/or to variations in the diet and consequently the microbiome of our experimental mice. In terms of tumor burden, we also measured the levels of M-Spike from recipient´s serum at the indicated time points, which did show significant tumor reduction by day 25 in the LCL161 + α-PD-1 cohort but by day 55, the MYXV-treated mice consistently showed further reductions in tumor burden (Figure 4C).

### Therapy with autologous BM *ex vivo* pre-loaded with MYXV and in combination with LCL161 and α-PD-1 was curative for a fraction of survivor mice that were re-challenged with fresh murine Vk12598 myeloma cells

**Figure 5:**
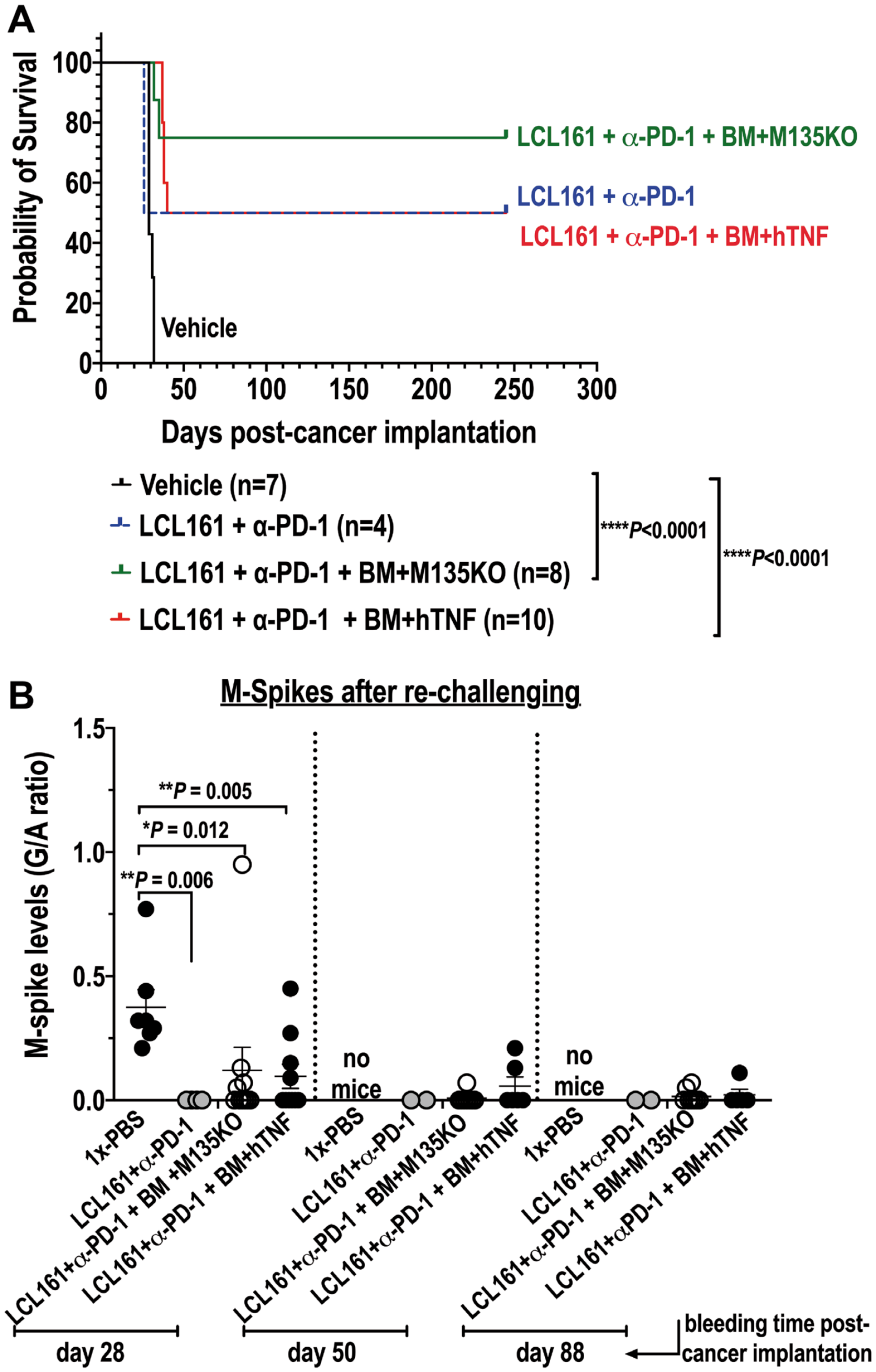
Combination therapy with auto-BM *ex vivo* loaded with either un-armed or hTNF armed MYXV with LCL161 and α-PD-1curative to mice seeded with BOR-resistant Vk12598 cells. (**A**) Shows survival curves of survivor mice re-challenged with Vk12598 multiple myeloma cells. (**B**) shows tumor burden (levels of M-spike) of those mice re-challenged with the Vk12598 myeloma cells. Survival curves (A) show the number of treated mice, survival (in days) from the beginning of re-challenging, and *P* values for log-rank Mantel-Cox survival test. Unpaired two-tailed *t* test *P* values are reported in (B). *P* values are reported as not statistically significant (ns) when *P* > 0.05 or statistically significant when ^*^ *P* < 0.05, ^**^ *P* 0.01, ^***^ *P* 0.001, ^****^ *P* ≤ 0.0001. No mice means that the mice at that particular time point have already succumbed to the disease.

The *in vivo* results shown in Figure 4B, clearly reveal greater therapeutic effects when recipient mice seeded with Vk12598 cells were treated with autologous BM loaded with MYXV (i.e., vMyx-M135KO or vMyx-hTNF) in combination with the Smac-mimetics compound LCL161 plus α-PD-1. Therefore, in order to determine whether or not these combination treatments can be curative for those survivor mice, 1 × 10^6^ Vk12598 cells, were implanted intravenously (i.v.) via the tail vein, in these survivor mice from the combinatorial treatment cohorts. Importantly, we found that 75% of those mice treated with LCL161+α-PD-1 followed by BM/ vMyx-M135KO and 50% of those mice treated with LCL161+α-PD-1 followed by BM/vMyx-hTNF survived for 245 days after re-challenging with murine Vk12598 cells (Figure 5). Furthermore, we found that mice that were first treated with LCL161+α-PD-1 followed by free M135KO or hTNF-armed MYXV eventually succumbed when re-challenged with fresh Vk12598 (data not shown). Interestingly, 50% of survivor mice (*n* = 4) that were originally treated LCL161+α-PD1 tolerated re-challenge with the same myeloma cells (Figure 5A), which is in agreement with data reported by Chesi and co-workers [12]. The levels of M-spikes shown in Figure 5B, correlates with the survival rates of four different cohorts. Together, our data support the notion that combination therapy with LCL161+α-PD-1 with BM *ex vivo* loaded with MYXV induces resistance to secondary challenge with Vk12598 cells.

## DISCUSSION

Treatment of MM is challenging because the disease inevitably relapses and becomes resistant to current standard therapies. Oncolytic viruses (OVs) have recently emerged as a potential therapeutic alternative to treat hematological malignancies like MM in preclinical models and/or clinical trials [22–26]. Therapeutic OVs can be used alone or as an adjunct therapy in combination with standard chemotherapeutic agents [22]. Depending on the types of tumors and their localization, OVs can be delivered intratumorally, systemically as free virus or after *ex vivo* loading onto carrier cells. Each of these delivery methods can impact the therapeutic outcomes. The systemic delivery of any free OV faces several therapeutic challenges including: (i) neutralization by circulating serum factors such as antibodies and complement, (ii) sequestration in secondary lymphoid organs like the spleen, or clearance in the liver, (iii) often targeting to vascular endothelial cells [27]. In contrast, using autologous BM leukocytes or PBMCs as carrier cells to deliver OVs to tumor sites has several advantages over the systemic delivery of free virus to selectively reach the tumor microenvironment. In the past, our lab has demonstrated that allogeneic or autologous BM grafts pre-loaded *ex vivo* with oncolytic MYXV can circumvent some of the disadvantages associated with the systemic delivery of free virus [6, 7, 13].

Another important advantage of using OVs is that they can activate the innate or adaptive immune system on their own or when combined with chemotherapeutic agents and/or ICIs [28, 29]. Furthermore, OVs can be genetically engineered to allow expression of therapeutic transgenes directly in the tumor bed to change the tumor microenvironment (TME) and to recruit anti-tumor immune cells. We recently demonstrated that autologous PBMCs *ex vivo* loaded with an engineered oncolytic MYXV expressing human TNF (vMyv-hTNF) was an effective therapeutic strategy against murine K7M2 osteosarcoma cells in a lung metastatic preclinical syngeneic murine model, whereas unarmed MYXV was ineffective [13].

In this study, we used the Vk*MYC MM model because it faithfully recapitulates the localization of the myeloma disease within the bone marrow as well as the clinical manifestation of the disease including bone damage (paralysis), renal failure [9, 12]. In addition to this, the Vk*MYC model allows determination of tumor burden (i.e., levels of circulating M-Spike protein) in the mouse serum using serum protein electrophoresis (SPEP) assay, which is routinely used in the clinic to track MM development and progression. Here we set out to investigate and compare the therapeutic effects of virotherapy with un-armed MYXV (i.e., vMyx-M135KO) with a recombinant MYXV armed with the human TNF (i.e., vMyx-hTNF), and also compare the anti-cancer efficacy of delivering virus systemically via retro-orbital (r.o.) injection of naked virus versus autologous BM *ex vivo* loaded with each of the above MYXV constructs alone or in combination with chemo/immunotherapy using the transplantable murine Vk*MYC multiple myeloma model.

Our *in vitro* studies demonstrate the capacity of MYXV to bind BOR-resistant Vk12598 multiple myeloma cells. Furthermore, we were able to show that MYXV can also infect these MM cells (Figure 1). Being able to access the levels of MYXV binding and virus infection of these MM cells is relevant taking in consideration that Vk12598 cells cannot be cultured for long periods of time *in vitro*. The fact that MYXV can infect and compromise the viability of these myeloma cells in a short period of time is relevant and suggest the potent oncolytic activity of MYXV against these BOR-resistant MM cells.

To determine if our *in vitro* results can be recapitulated *in vivo,* bortezomib-resistant Vk12598 MM cells were seeded in immunocompetent C57BL/6 mice and then transplanted with auto-BM cells that had been *ex vivo* loaded with either unarmed vMyx-M135KO or transgene-armed vMyx-hTNF virus constructs. Our *in vivo* results demonstrated that under these therapeutic conditions, not only was the onset of the disease delayed, but also, overall survival rates increased in a fraction of immunocompetent C57BL/6 mice pre-seeded with Vk12598 myeloma cells as shown in Figure 2B. However, we could not establish statistically significant differences upon transplantation with auto-BM *ex vivo* loaded with either of the described virus constructs, suggesting the therapeutic effects emanate from the virus backbone, not the TNF transgene tested. These *in vivo* results although were unpredicted based on our *in vitro* results and the fact that we had expected higher therapeutic effects of the armed MYXV construct with a pro-inflammatory cytokine like TNF, as had been observed in a syngeneic lung metastatic osteosarcoma model [13]; however our *in vivo* results were not entirely surprising since TNF has been reported by others to promote IL-6, a propagating factor for MM [30, 31] that suppresses apoptosis of MM by activating the NF-κB-pathway [32]. This is in contrast of recently published meta-analysis demonstrating the protective effect of TNF against MM risk in the dominant model and allele analysis [33]. These published inconsistences in regards the effects of TNF on myeloma progression or inhibition prompted us to investigate the therapeutic effects of this cytokine in the context of oncolytic virotherapy with MYXV. Taken together, we observed some therapeutic effects in mice seeded with MM specially when auto-BM cells were *ex vivo* loaded with the TNF transgene as compared to treatment with free vMyx-hTNF (Figure 2B).

Another important aspect of our study is that the means of systemic delivery of oncolytic MYXV matters because by using BM autographs *ex vivo* loaded with MYXV, superior therapeutic effects (i.e., 50% overall survival) were observed as compared to the systemic delivery of the free virus, which was ineffective compared to the delivery using autologous BM armed with the virus (i.e., 10–20% overall survival rates) (Figure 2B). In principle, the observed improvement of survival rates in this mouse model with established myeloma when treated with BM/MYXV can be attributed to multiple reasons: (i) an increase in the dosage of oncolytic MYXV delivered to the tumor microenvironment in the BM resulting in more effective oncolysis; (ii), MYXV together with the carrier BM cells, can trigger a native or adaptive immune response resulting in superior anti-tumor effects; or (iii) a combination of both (i) and (ii). In contrast, the systemic delivery of free virus can encounter physiological barriers such as virus neutralization by antibodies, antiviral cytokines and tissue-resident macrophages, resulting in a rapid clearance of the virus from the circulation before it reaches the cancerous target in the bone marrow. Also, the non-specific uptake of the virus by the spleen and/or liver, for further clearance [34], and the poor virus escape from the vascular compartment [35]. Also, even though only a small fraction of mice survived this regimen, all of the survivors became resistant to a second challenge with fresh Vk12598 cells (Figure 2B, 2C), suggesting that these re-challenged mice have become resistant against aggressive and drug refractory MM cells, and importantly these results suggest that the re-challenged mice have developed acquired anti-tumor immunity to the myeloma cells.

In the past, others have shown that the oncolytic reovirus synergizes with the standard-of-care MM drug bortezomib against MM using the Vk12598 murine model of MM [22]. This result was surprising considering that Vk12598 cells are insensitive to bortezomib (BOR) treatment. However, the authors found that BOR augmented reovirus replication in MM cells and in tumor-associated endothelial cells, increasing viral delivery and consequently stimulation of cytokine release, apoptosis, immune activity and reduction of anti-tumor immunosuppression [22]. Therefore, we hypothesize that combination therapy with MYXV and chemo/ immunotherapeutic agents might also increase survival and eradicate myeloma *in vivo*. To test this hypothesis, mice bearing MM tumors were treated with the alkylating agent Cy in combination with autologous BM *ex vivo* loaded with MYXV. As mentioned before, Cy is one of the forefront chemotherapeutic drugs used to treat multiple myeloma specially in combination with other chemotherapeutic agents [36]. Unfortunately, the anti-myeloma effects of this alkylating agent by itself are not sustained for a long period of time and eventually the disease relapses [36]. For our *in vivo* experiments, mice were seeded with myeloma cells for 3 weeks, which assures the establisment of later-stage disease. Then mice were treated with Cy twice, each dose 1 week apart in order to induce minimal residual disease (MRD). After this, mice were treated with auto-BM *ex vivo* loaded with unarmed vMyx-M135KO or with armed vMyx-hTNF. As shown in Figure 3B, we could not observe any synergistic effect with this combination therapy. Although the progression of the disease was delayed, no statistically significant differences were observed among the cohorts treated with Cy alone (28% overall survival) versus Cy followed by BM/ vMyx-M135KO (50% overall survival) or BM/vMyx-hTNF (25% overall survival). Nevertheless, the therapeutic benefits with treatment including Cy followed by BM/vMyx-M135KO were modestly superior than that one using Cy and BM/vMyx-hTNF, implying that the TNF transgene might have actually interfered with effacacy. One possible reason for this observation is that combination of Cy and MYXV armed with hTNF might have induced undesirable side effects, including cytotoxicity towards T lymphocytes. In addition, it has been reported that TNF along with IL-6, IL-1β synergizes with receptor activator of nuclear factor κB ligand (RANKL) to induce osteoclastogenesis in MM [37, 38].

Finally, we explored combination therapy, including the LCL161 Smac-mimetics compound plus the ICI α-PD-1 followed by transplantation with BM leukocyte carrier cells armed with MYXV. The Smac-mimetics compound LCL161 completed a phase 1–2 clinical trials for MM. This compound acts by debulking human osteoclasts and compromising their viability [39]. On the other hand, the use of α-PD-1 as monotherapy against MM has demonstrated lack of activity in clinical trials [40]. In 2016, Chesi and co-workers demonstrated outstanding therapeutic benefits with the combination of LCL161 plus α-PD1 in the transplantable Vk*MYC murine MM model [12]. In fact, the authors demonstrated synergistic effects of this combination therapy and acquisition of immunity by survivor mice that were re-challenged with Vk*MYC cells. We noted dramatic M-Spike reductions in the LCL161/α-PD1 cohort by 25 days (Figure 4C) and we did observe that the combination of LCL161/α-PD-1 followed by autologous transplantation with BM cells *ex vivo* loaded with either vMyx-M135KO or vMyx-hTNF, had additional therapeutic benefits in mice seeded with Vk12598 cells (i.e., overall survival with vMyx-M135KO = 75% vs. vMyx-hTNF = 60%) (Figure 4). Furthermore, results shown in Figure 5 suggest that survivor mice might have acquired immunity against re-challenging with Vk12598 cells.

Together, we show promising results in terms of therapeutic benefits of delivering oncolytic MYXV via carrier cells from autologous BM transplants, both alone or in combination with LCL161 and α-PD-1 against drug-resistant MM cells *in vivo*. To our knowledge, these are the first results showing therapeutic benefits of oncolytic MYXV to control and even eradicate established drug-resistant MM cells in a preclinical murine model that has previously shown excellent concordance with predicting clinical efficacy in human MM patients. Although in this study, we have demonstrated that autologous bone marrow leukocytes can efficiently be packed with an oncolytic virus like MYXV, resulting in therapeutic effects against drug resistant MM cells, future studies should be focused on understanding the mechanisms by which oncolytic virotherapy with MYXV alone or in combination with chemotherapeutic/immunotheraputic agents results in cancer-free or cancer-controlling outcomes in the pre-clinical murine Vk*MYC model. In the past, using a different MM murine model we have also demonstrated that certain autologous or allogeneic bone marrow or PBMCs derived leukocytes are better carriers of MYXV into the tumor microenvironment of MM [27, 28]. Therefore, it will be important to identify potential autologous BM leukocyte carriers of MYXV using the Vk*MYC model.

## MATERIALS AND METHODS

### Cells and viruses

The murine bortezomib resistant Vk12598 was a kind gift of Drs. Marta Chesi (Ph.D.), and P. Leif Bergsagel (M.D.) (Mayo Clinic, Arizona). These cells were used to perform *in vitro* and *in vivo* assays.

Three myxoma virus constructs were used in this study: vMyx-M093L-Venus (wild-type MYXV that expresses Venus-tagged M093 protein as a virion component), vMyx-M135KO (an un-armed and attenuated recombinant MYXV, in which the M135 gene has been deleted and the green fluorescent protein (GFP) has been inserted under a poxvirus synthetic early/late promoter) [41] and vMyx-hTNF (GFP-expressing knock in of the human TNF gene inserted into the M131 gene) [13, 41, 42].

### Myxoma virus binding and infection of Vk12598 myeloma cells

For virus binding, Vk12598 MM cells were incubated with vMyx-M093L-Venus at an MOI = 10 for 1 hour at 4°C to allow virus particles binding to cells. Unbound virus was washed twice with 1x-PBS supplemented with 5% FBS. For virus infection, vMyx-M135KO expressing the GFP protein driven by an early/late viral promoter was incubated at MOI = 10 with Vk12598 cells for 1 hour at 37°C to allow virus adsorption. After these, cells were incubated at 37°C for 18 hours to allow virus infection. Virus binding to the Vk12598 MM (CD138^+^CD220^−^) cells or virus infection of these myeloma cells was assessed using flow cytometry.

### Flow cytometry analysis

To isolate spleen and BM, mice were euthanized by CO_2_ inhalation. Once collected, from the spleen a single-cell suspension was obtained by mechanical disruption between frosted glass slides. On the other hand, BM cells were collected by flushing out ilium, femur, tibia, humerus, and radius with 1x-PBS. On average, about 1.0 × 10^8^ of BM nucleated cells were obtained from each mouse. These samples were used to perform virus binding and infection assays, as well as to determine the number of MM cells remaining after virus infection. For this latter assay, Vk12598 (CD138^+^) cells mock treated (i.e., without adding the virus) or infected with vMy-M135KO were labeled with near-IR-live/dead stain (Invitrogen). To assess of percentage of MM cells (B220^−^CD138^+^) in bone marrow and spleen and to quantify levels of virus binding and infection of CD138^+^CD220^−^ MM cells and the cell viability upon virus infection of these myeloma cells flow cytometry was used according to described protocols [9]. Fluorescently labeled monoclonal antibodies such as B220-APC (clone RA3-6B2) and CD138-BV605 (clone 281-2) were from Biolegend. For each experimental condition a minimum of 300,000 events were acquired on a LSRFortessa cytometer (BD Biosciences) and acquired data were analyzed using the FlowJo software.

### Administration of chemotherapeutic drugs and α-PD-1 check point inhibitor

For drug dosage and route of administration, we followed reported methods [12]. In brief, cyclophosphamide (Cy) (Tocris Bioscience) was used at a final concentration of 100 mg/Kg and administrated intraperitoneally (i.p.) twice, each dose one week apart. When LCL161 (Chemietek) was used, 50 mg/Kg LCL161 was administrated via the oral gavage (o.g.) route. The checkpoint inhibitor α-PD-1 (BioXCell) was administrated intraperitoneally (i.p.) at a final concentration of 10 mg/Kg. One week after cancer implantation, both LCL161 and α-PD-1 were administrated on days 1, 4, 8, 11 as described in Figure 4A.

### *In vivo* mouse studies

All animal experiments were performed under the Institutional Animal Care and Use Committee (IACUC) approval (no. 171543R) of Arizona State University and conformed to all regulatory standards. For *in vivo* autologous BM-derived leukocyte transplantations, we used, 8-weeks old C57BL/6 female or male mice, (Charles River laboratories, Wilmington, MA) as donors or recipients, which were matched according to gender and age. First, immunocompetent C57BL/6 recipient mice were implanted intravenously (i.v.) via tail vein with 1.0 × 10^6^ BOR-resistant Vk12598 cells. One week after myeloma implantation, mice were randomized to six groups or cohorts (i.e., a minimum of 7 or 10 mice per group, unless other number specified) and treated as follows: No BM transplant but 1x-PBS vehicle control (cohort I); 2.0 × 10^6^ autologous BM cells alone (cohort II); 1.0 × 10^7^ focus forming (i.e. infectious) units (ffu) of free un-armed vMyx-M135KO virus construct (cohort III); 2.0 × 10^6^ BM cells *ex vivo* pre-loaded with un-armed vMyx-M135KO at a multiplicity of infection (MOI) of 10, (cohort IV); 1 × 10^7^ ffu of free transgene-armed vMyx-hTNF virus construct (cohort V); 2.0 × 10^6^ autologous BM cells *ex vivo* treated with transgene-armed vMyx-hTNF virus construct at MOI of 10 (cohort VI). Each treatment was delivered via retro-orbital (r.o.) route. At the onset of morbidity (i.e., hind limb paralysis, hunched position, weight loss, labor breathing, or at the end of the study, mice were euthanized via asphyxiation with CO_2_, as per institutional guidelines. Survival was assessed 100 days following cancer implantation. For re-challenge experiments, MYXV-treated mice from transplant cohorts that survived 100 days, post-cancer implantation were re-infused with fresh 1.0 × 10^6^ Vk12598 cells intravenously (i.v.) via tail vein. Mice that survived more than 100 days post cancer re-challenged were considered cured and had acquired immunity to the myeloma.

For *in vivo* experiments using the chemotherapeutic compound cyclophosphamide (Cy), mice were injected with 1 × 10^6^ Vk12598 cells and the disease was allowed to be established for 3 weeks. Then mice were treated twice with Cy in order to debulk the tumor burden. It is known that Cy induces a transient anti-myeloma response [36]. Then mice were treated as follows: 1x-PBS, vehicle control (cohort I), cyclophosphamide alone (cohort II), Cy followed by treatment with 2.0 × 10^6^ of autologous BM cells *ex vivo* loaded with un-armed vMyx-M135KO at a multiplicity of infection (MOI) of 10, (cohort III), or 2.0 × 10^6^ of autologous BM cells *ex-vivo* loaded with vMyx-hTNF at MOI of 10 (cohort IV). Likewise, combination therapy with 50 mg/Kg LCL161 and 10 mg/Kg α-PD-1, as described before, was followed by autologous BM transplantation + MYXV. In brief mice were treated as follows: 1x-PBS (cohort I); LCL161 + α-PD-1 (cohort II); LCL161 + α-PD-1 + free vMyx-M135KO (cohort III); LCL161 + α-PD-1 + free vMyx-hTNF (cohort IV); LCL161 + α-PD-1 + BM + vMyx-M135KO (cohort V); LCL161 + α-PD-1 + free vMyx-hTNF (cohort VI).

### Assessment of myeloma burden using serum protein electrophoresis (SPEP)

Blood was collected from mice periodically by cheek bleeding. About 100 μL of whole blood were collected into microtainer tubes (BD Biosciences), allowed to coagulate at room temperature, and spun for 10 minutes at 2,300 × *g*. Sera were diluted 1:2 in normal saline buffer and analyzed on a QuickGel Chamber apparatus using pre-casted QuickGels (Helena Laboratories) according to the manufacturer’s instruction. Densitometric analysis was then performed using the clinically certified Helena QuickScan 2000 workstation which allows a precise quantization of the various serum fractions, including the measurements of gamma/albumin (G/A) ratio. A G/A ratio between 0.5–2.0 corresponds to predominant M-spikes and suggest not only good multiple myeloma engraftment but also tumor response to treatments in similar factions as is done clinically [10].

### Statistics

Values are represented as means ± SD for at least two or three independent experiments.

Kaplan-Meier analysis of mouse survival was performed with GraphPad Prism 8 software (La Jolla, CA, USA), and log-rank (Mantel-Cox) test was performed to compare survival curves and to perform statistical analysis. Statistical comparison between two groups was conducted using the 2-tailed Student’s *t*-test. Animals were assigned to treatment groups (Cohorts) randomly and the number of animals in each treatment group is reported in the figures. *P* values are reported as follows: no significant (ns) *P* > 0.05, ^*^ *P* < 0.05, ^**^ *P* 0.01, ^***^ *P* 0.001, ^****^ *P* 0.0001.

### Study approval

All animal work was conducted under the approval of Arizona State University Institutional Animal Care and Use Committee (IACUC) (no. 171543R) in accordance with federal, state, and local guidelines.
